# Association between health-related social media use and E-cigarette use among current cigarette users: the roles of anti-tobacco messages and harm perception

**DOI:** 10.1186/s12889-024-18756-8

**Published:** 2024-05-10

**Authors:** Luxi Zhang, Sha Sarah Qiu, Song Harris Ao, Xinshu Zhao

**Affiliations:** 1 Department of Communication / Institute of Collaborative Innovation, University of Macau, Macau, China; 2 Department of Communication / Institute of Collaborative Innovation / Center for Research in Greater Bay Area, University of Macau, Macau, China

**Keywords:** Health-related social media use, Current cigarette users, E-cigarette use, Online anti-tobacco messages exposure, Harm perception of e-cigarettes

## Abstract

**Background:**

The popularity of e-cigarettes is on the rise among current cigarette users. Therefore, there are concerns about their health implications. This study examined the impact of health-related social media use on e-cigarette use among current cigarette users. It assesses the mediating influence of online anti-tobacco messages and the moderating role of the harm perception of e-cigarettes.

**Methods:**

This study was focused on 563 current cigarette users from the 2022 Health Information National Trends Survey (HINTS). Three tasks were performed: (1) assessing the direct and indirect impacts of health-related social media use on e-cigarette use among current cigarette users, (2) exploring the mediating role of exposure to online anti-tobacco messages, and (3) examining the moderating influence of e-cigarette harm perception on the path from anti-tobacco messages to e-cigarette use.

**Results:**

Health-related social media use was positively associated with current cigarette users’ e-cigarette use directly (bp = 0.183, *p* < .01) and indirectly through exposure to online anti-tobacco messages (bp = 0.023, 95% CI: [0.001, 0.051]). Harm perception of e-cigarettes moderated the relationship between online exposure to anti-tobacco messages and e-cigarette use (bp=-0.467, *p* < .01). The relationship appeared weaker for individuals who perceived greater harm from e-cigarettes.

**Conclusions:**

Health-related social media use positively correlates with e-cigarette use among current cigarette users through exposure to online anti-tobacco messages. The perceived harm of e-cigarettes moderates this indirect path. These findings have implications for public health interventions aimed at smoking cessation.

## Introduction

The global prevalence of tobacco products has led to increasing rates of smoking-related diseases and deaths, making smoking cessation a crucial priority in the field of public health [1]. The current landscape of tobacco use presents new challenges and opportunities. For instance, there has been a decrease in the number of cigarette users who are addicted to traditional cigarettes in this decade [2]. Additionally, the availability of various alternative nicotine-delivery devices allows adults who smoke a range of options [3].

The e-cigarette, or electronic nicotine delivery system (ENDS), is a new nicotine product [4–6] that has become increasingly popular in the United States [7, 8]. Initially marketed as smoking cessation products [9], the effectiveness of e-cigarettes in aiding smoking cessation remains a topic of controversy [10, 11]. Specifically, the dual use of cigarettes and e-cigarettes is an emerging phenomenon [12], as substantiated by multiple studies [13–16], that has been found to contribute to the prolonged use of combustible tobacco products. Moreover, most studies [17, 18] indicate that current cigarette users are more inclined to engage in the use of e-cigarettes, which raises our concerns regarding the factors contributing to this transition.

Health-related social media are presented on social media platforms for various health-related purposes and involve the active participation of health institutions, professionals, and the public in leveraging digital channels to address health issues and promote well-being [19]. Unprecedented growth in social media use has revolutionized the way that individuals access and share health-related information, which may offer a unique avenue for users to engage with a broader range of health topics, including smoking cessation [20, 21].

Notably, there are apparent differences between general social media use and specific health-related social media use (HSMU). First, much health-related content is generated by public health government or experts’ social media accounts; these accounts convey a greater sense of professionalism and credibility than general social media accounts [22]. Second, users’ search intentions for health-related social media are usually more focused on their health concerns [23]. These disparities in content and user intentions underscore the heightened importance of health-related social media platforms within the realm of public health, similar to the realm of tobacco control.

E-cigarettes have often been portrayed on social media platforms as a means to relieve cravings or reduce cigarette consumption among those attempting to quit smoking [24, 25]. However, the literature addressing the effects of HSMU on cigarette and e-cigarette use (ECU) remains limited [26]. Further exploration is warranted to understand the mechanisms underlying the relationship between HSMU and e-cigarette consumption among current cigarette users to bridge this research gap. Then we can come out with our first research question:

### RQ1

Can health-related social media use (HSMU) affect current cigarette users’ turn to use e-cigarettes?

### Theory framework

In our research context, we anchor this study to Harm Reduction Theory (HRT). HRT acknowledges the persistence of unhealthy behaviors and centers on minimizing their associated risks rather than pursuing complete elimination, resulting in a focus on reducing harm rather than imposing immediate cessation of these behaviors [27]. Originally devised to address the multifaceted harms associated with drug abuse [28–30], HRT has evolved to encompass broader health concerns, including alcohol abuse [31] and tobacco use [32]. At its core, this theory underscores the importance of balancing risks and benefits, recognizing that absolute cessation may not always be immediately achievable. In this context, e-cigarettes, while not without risks, can be viewed as a harm reduction strategy compared to traditional cigarettes, aligning with the core principles of HRT.

### Formulating research questions and hypotheses

Anti-tobacco campaigns and advertisements are widely used to raise awareness of the risks associated with tobacco product use, with the aim of reducing smoking [33]. Previous research suggests that, due to the limited resources of tobacco control programs, optimal effectiveness can be achieved by focusing efforts on specific objectives, such as promoting smoking cessation among adult cigarette users [34–36]. Additionally, previous studies have demonstrated that anti-tobacco campaigns aimed at the general population are also more likely to impact adults who smoke cigarettes than nonsmokers [34, 37].

With the advancement of the internet and social media platforms, online anti-tobacco messages have emerged and evolved, potentially amplifying their exposure among cigarette users. Studies have shown that cigarette users are more likely to encounter tobacco-related messages on social media, including both pro- and anti-tobacco messages [38].

Furthermore, Ahadzadeh, Sharif [23] underscored the propensity of health-related social media platforms to attract health-conscious individuals who use them for health purposes. Because the algorithmic mechanisms of social media platforms can contribute to the creation of “echo chambers” [39], current cigarette users who engage with health-related social media content about smoking risk may be more inclined to encounter anti-tobacco messages. Consequently, they may have a greater likelihood of being exposed to anti-tobacco messages on the internet [40], including social media, websites, and other online platforms.

Despite anti-tobacco messages generally aiming to discourage the use of all kinds of tobacco products, research shows that the majority of studies on the effects of anti-tobacco messages primarily concentrate on cigarettes [41]. This research preference may suggest a predominant focus on anti-cigarette messaging in both online and offline contexts [40]. Additionally, some stakeholders associated with e-cigarettes may seek to promote them for smoking cessation while downplaying their harmful effects on social media [42, 43]. This status quo could lead current cigarette users to perceive traditional cigarettes as more harmful than e-cigarettes.

To our knowledge, the influence of online anti-tobacco messages on current cigarette users transitioning to ECU remains an underexplored area in current research. A pioneering study [38] investigated the association between self-reported engagement with anti-tobacco messages on social media and subsequent ECU. However, the findings showed no significant correlation between such engagement and ECU.

In the early days of e-cigarettes, public belief that e-cigarettes are healthier than traditional cigarettes was expected, and people were given reasons to use e-cigarettes to quit smoking [44]. However, as studies on e-cigarettes continue to advance, there has been growing concern about the potential harm of e-cigarettes and their impact on public health [45–47]. Moreover, the emergence of two critical public health concerns, namely Electronic Cigarette, or Vaping, Product Use-Associated Lung Injury (EVALI) and coronavirus disease 2019 (COVID-19), in the United States has made an increasing number of people concerned about the harm caused by e-cigarettes [48, 49].

Harm perception might have moderating effects on current cigarette users’ decisions regarding ECU. Multiple studies have demonstrated that those who perceive e-cigarettes as less harmful than traditional cigarettes are more likely to use e-cigarettes [50–53]. The difference in the perceived harm of e-cigarettes across population samples (e.g., daily users, non daily users, and triers) has been proven in previous studies [54]. These findings suggest that current cigarette users with a heightened sense of harm associated with e-cigarettes may be less likely to use e-cigarettes.

Based on the literature reviews presented above, the following hypotheses and research questions emerge:

#### H1

HSMU is positively associated with online anti-tobacco message exposure (OAT). The greater the HSMU is, the greater the OAT among current cigarette users.

#### RQ2

How does OAT affect e-cigarette use (ECU) among current cigarette users?

#### RQ3

Does exposure to online anti-tobacco messages mediate the relationship between HSMU and ECU among current cigarette users?

#### H2

Harm perception of e-cigarettes (HPE) has a negative moderating effect on the association between OAT and ECU among current cigarette users. As the HPE increases, the positive impact of OAT on ECU decreases.

## Materials and methods

### Data source and sampling

The data for this study were obtained from the 2022 Health Information National Trends Survey (HINTS) 6 dataset (https://hints.cancer.gov/) and were collected between March and November 2022. Participants were drawn from this comprehensive survey designed to investigate various health-related behaviors and attitudes among individuals across the United States, and the dataset comprised a total of 6,252 surveys. To ensure nationally representative findings, we applied gender weights (50.7% female and 49.3% male), age weights (35.7% 18–44 years, 25.4% 45–64 years, and 16.8% 65 years and above), and race/ ethnicity weights (57.8% non-Hispanic White, 12.1% non-Hispanic Black, 18.7% Hispanic, 6% non-Hispanic Asian and 5.4% non-Hispanic Other) from the American Community Survey (ACS), resulting in a decrease in the number of valid participants to 5268. Based on the weighted survey sample, we identified a subset focusing specifically on current cigarette users (participants who reported having smoked at least 100 cigarettes in their lifetime and now smoke cigarettes every day or someday), resulting in a final sample of 563 participants.

### Demographic characteristics

We selected the following sociodemographic variables for the study: gender, age, education level, marital status, and race/ethnicity. Age was collapsed into three groups and coded as follows: (1) 18–44 years, (2) 45–64 years, and (3) 65 years and above. Gender was coded as follows: (0) female and (1) male. Education level was collapsed into seven groups and coded as follows: (1) less than 8 years, (2) 8 through 11 years, (3) 12 years or completed high school, (4) post-high school training other than college, (5) some college, (6) college graduate, and (7) postgraduate. Marital status was coded into two categories: (1) non-single or (0) single. Race/Ethnicity was coded as follows: (1) non-Hispanic White, (2) non-Hispanic Black, (3) Hispanic, (4) non-Hispanic Asian, and (5) non-Hispanic Other.

### Study variables

The dependent variable, *e-cigarette use*, was derived from two questions: (1) Have you ever used an e-cigarette? (yes/no) and those who answered “yes” were asked the following question: (2) Do you now use an e-cigarette every day, some days, or not at all? (every day/some days/not at all). To align with the previous study on defining the prevalence of ECU [55–57], we recoded this variable by amalgamating the two questions to assess usage frequency, ranging from 0 to 3. Here, 0 indicated never use, 1 indicated past use, 2 indicated occasional use, and 3 indicated daily use [26, 55].

*Health-related social media use* [58] was the sum of the responses to four questions: In the past 12 months, how often did you do the following? (Shared personal health information on social media/ shared general health-related information on social media/interacted with people who have similar health or medical issues on social media or online forums/watched a health-related video on a social media site). Each item ranged from 1 (never) to 5 (almost every day) (*α* = 0.85) and was linearly transformed into a 0–1 scale. The constructed variable health-related social media use ranged from 1 to 5.

Following previous research [59], we assessed message exposure using one question, “During the past 3 months, have you noticed or heard any anti-tobacco messages (including cigarette and e-cigarette) in any of the following places?” *Online anti-tobacco message exposure* was the sum of 2 options (on social media/ other websites or online sources), with the response of “yes” coded as 1 or “no” coded as 0.

We assessed the *harm perception of e-cigarettes* [45, 60] by asking, “Compared to smoking cigarettes, would you say that using e-cigarettes that contain nicotine is?” The answer options were recoded as follows: “ (1) Must less harmful”, “ (2) Less harmful”, “ (3) Just as harmful”, “ (4) More harmful”, “ (5) Much more harmful”. Higher scores indicated greater perceived harm associated with e-cigarettes.

### Statistical methods

The data analysis was conducted using SPSS version 26. Initially, a bivariate Pearson correlation was performed to explore the associations between HSMU, OAT, HPE, and ECU. Subsequently, SPSS PROCESS was employed to investigate two aspects. First, we examined the potential mediating role of OAT in the relationship between HSMU and ECU with PROCESS Model 4. Second, the moderation effect of the HPE on the path from OAT to ECU was analyzed with PROCESS Model 14. A confidence interval (*CI*) of 95% was applied throughout the analysis, employing bootstrapping with 5000 iterations using the bias-corrected method.

Furthermore, in this study, the percentage coefficient (*bp*) was incorporated to complement the well-known *β* indicator, providing a comprehensive estimation of the effect size [43, 61–63]; *bp* represents a *b* coefficient when the dependent and independent variables are linearly transformed to a percentage scale ranging from 0 to 1.

## Result

### Demographic and participants’ characteristics

As shown in Table 1, the participants’ age distribution showed that among current cigarette users, 44% are from 45 to 64 years, and the age above 64 occupied only 16.1%. Gender distribution showed that males (52.7%) were little more than females (47.3%), and race/ethnicity was predominantly non-Hispanic White (57.2%).

Regarding marital status, more than half (56.1%) of the respondents reported being single. Approximately 25.8% had graduated from college or higher. The above demographic results almost complied with smoking adults statistics reported by the CDC (https://www.cdc.gov/). Of the OAT respondents, 20.3% reported being exposed to social media, and 12.0% reported being exposed to other websites or online sources. Notably, the mean score of HPE was 3.24, ranging from 1 to 5, representing a range from much less harmful to much more harmful.

**Table 1:** Descriptive statistic (n = 563)

Dependent variable		Natural scale	Percentage scale
E-cigarette use (ECU) (M ± SD)		0.75 ± 0.84	0.25 ± 0.28
**Independent variable** (α = 0.85)			
Health-related social media use (HSMU) (M ± SD)		1.58 ± 0.72	0.14 ± 018
**Mediating variable**			
Online anti-tobacco messages exposure (OAT) (n.%)			
Social media	Yes	114 (20.3)	N/A
	No	445 (79.1)	N/A
Other websites	Yes	67 (12.0)	N/A
	No	493 (87.4)	N/A
**Moderating variable**			
Harm perception of e-cigarettes (HPE) (M ± SD)		3.24 ± 1.05	0.56 ± 0.26
**Sociodemographic controls**			
**Age** (year, M ± SD)			
18–44 years		224 (39.8)	N/A
45–64 years		248 (44.0)	N/A
65 years and above		91 (16.1)	N/A
**Gender** (n. %)			
Female		267 (47.3)	N/A
Male		297 (52.7)	N/A
**Race/ Ethnicity** (n. %)			
Non-Hispanic White		322 (57.2)	N/A
Non-Hispanic Black or African American		90 (15.9)	N/A
Hispanic		80 (14.2)	N/A
Non-Hispanic Asian		23 (4.2)	N/A
Non-Hispanic Other		48 (8.5)	N/A
**Marital status** (n. %)			
Non-single		245 (43.4)	N/A
Single		316 (56.1)	N/A
**Education** (n.%)			
Less than 8 years		14 (2.5)	N/A
8 through 11 years		53 (9.5)	N/A
12 years or completed high school		134 (23.8)	N/A
Post high school training other than college		65 (11.5)	N/A
Some college		148 (26.3)	N/A
College graduate		96 (17.0)	N/A
Postgraduate		50 (8.8)	N/A

SD: standard deviation; M: mean; N/A: not applicable

*Note* The percentage scale indicates that all variables are linearly transformed to a percentage scale ranging from 0 to 1.

### Relationships among key variables

The bivariate correlations among the key variables of the study are presented in Table 2, revealing significant associations among HSMU, OAT, HPE, and ECU (ranging from − 0.313 to 0.183, *p* < .01).

**Table 2:** Zero-order Pearson correlations (*n* = 563)

Variables	1	2	3	4	5	6	7	9	10
1.E-cigarette use (ECU)	1								
2.Health-related social media use (HSMU)	0.158^**^	1							
3.Online anti-tobacco messages exposure (OAT)	0.183^**^	0.188^**^	1						
4.Harm perception of e-cigarettes (HPE)	− 0.313^**^	− 0.069	− 0.107*	1					
5.Age	− 0.257^**^	− 0.270^**^	− 0.199^**^	0.126^*^	1				
6.Gender	0.018	− 0.064	− 0.041	− 0.115^*^	0.075	1			
7.Race	− 0.076	0.085^*^	− 0.083	0.053	− 0.076	0.064	1		
8.Marital status	− 0.057	− 0.003	0.066	− 0.024	− 0.066	0.092^*^	0.015	1	
9.Eductaion	0.025	0.153**	0.015	− 0.060	− 0.090*	− 0.042	0.072	0.048	1

**Fig. 1:**
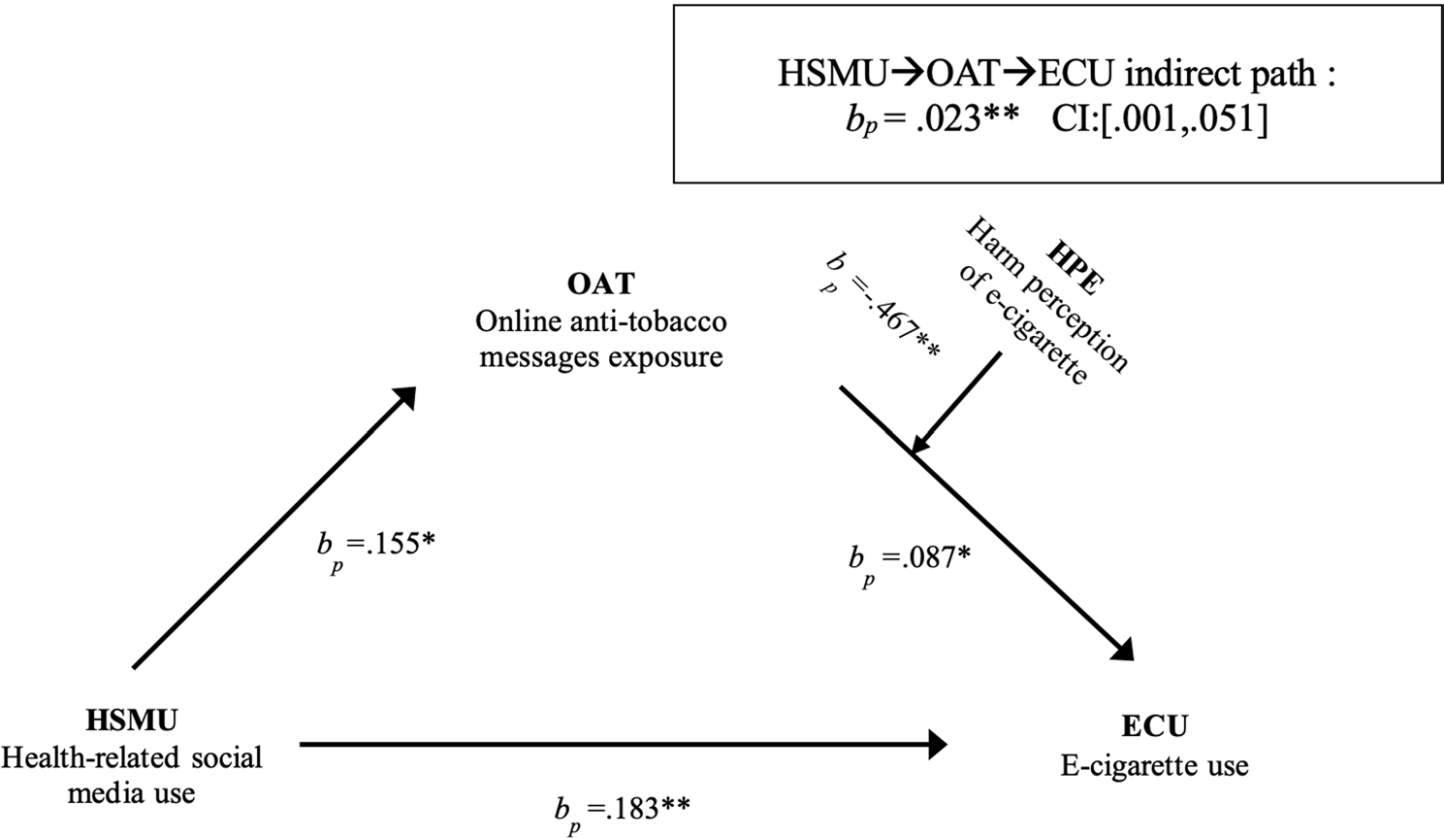
Effects of HSMU on the ECU, mediated by OAT, moderated by HPE. Path indicators are percentage coefficients (bp). *p < .05; **p < .01; ***p < .001. HSMU: health-related social media use; ECU: e-cigarette use; OAT: online anti-tobacco messages exposure; HPE: harm perception of e-cigarettes

As illustrated in Table 3; Fig. 1, a significant direct association was detected between HSMU and ECU (*bp* = 0.183, *p* < .01), which answered **RQ1**. Additionally, HSMU had a statistically significant positive relationship with OAT (*bp* = 0.155, *p* < .05), with ECU controlled for. The results supported **H**1. OAT also had a statistically significant and positive relationship with ECU (*bp* = 0.087, *p* < .05). This result answered **RQ2** by indicating that OAT positively impacts ECU among current cigarette users. The findings in Table 3 also support the indirect relationship between HSMU and ECU through OAT (*bp* = 0.023, *CI*: [0.001, 0.051]), which answered **RQ3**. OAT mediated the relationship between HSMU and ECU.

**Table 3:** Summary of mediation and moderation effects (n = 563)

Mediation pathway	bp	β	SE	95% CI
*a* path: HSMU→OAT	0.155*	0.039*	0.073	[0.011, 0.298]
*b* path: OAT → ECU	0.087*	0.261*	0.040	[0.009, 0.165]
a*b path: HSMU→ OAT →ECU	0.023**	0.017**	0.013	[0.001, 0.051]
*d* path: HSMU →ECU	0.183**	0.137**	0.068	[-0.049, 0.316]
**Moderation pathway**				
OATxHPE →*b* path	− 0.467**	− 0.350**	0.175	[-0.811, − 0.123]

*p < .05; **p < .01; ***p < .001

*bp*: percentage coefficients; *β*: Standardized beta; SE stands for standard error; CI stands for confidence interval

HSMU: health-related social media use; OAT: online anti-tobacco messages exposure; ECU: e-cigarette use; HPE: harm perception of e-cigarettes

All model controlling for age, gender, race/ethnicity, marital status, education; *a* path controlling for ECU

**Fig. 2:**
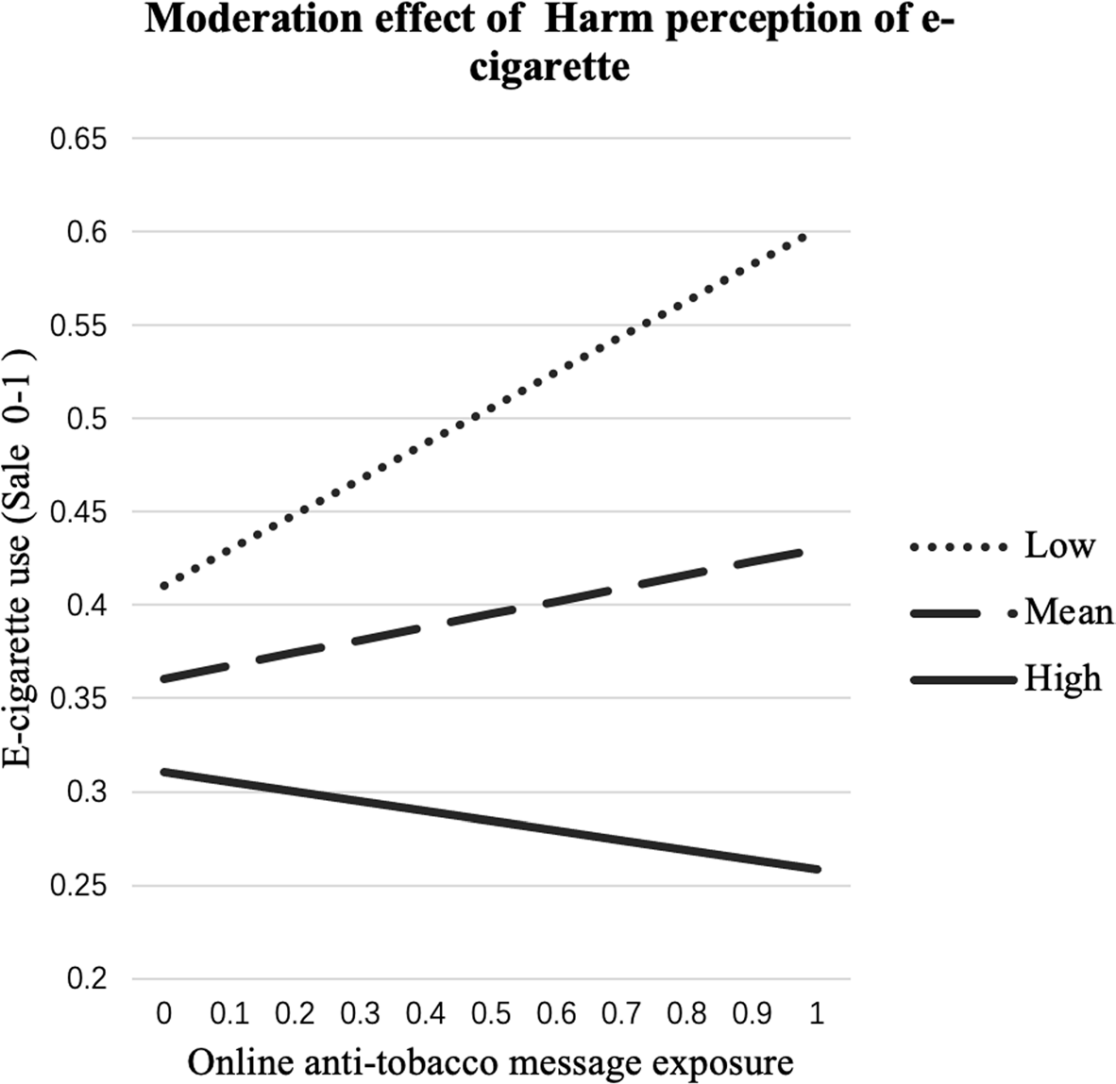
OAT→ECU effect moderated by HPE. Vertical and horizontal axes are both on 0 ~ 1% scales. OAT: online anti-tobacco messages exposure; ECU: e-cigarette use; HPE: harm perception of e-cigarettes

Furthermore, the results in Table 3; Fig. 2 indicate a moderating effect of HPE on the path from OAT to ECU (*bp* = − 0.467, *p* < .01), supporting **H2**.

## Discussion

The primary objective of this study was to explore the mechanisms and conditions underlying the connection between HSMU and ECU among current cigarette users. Through a focused investigation, our findings illuminate a multifaceted correlational relationship among these factors, offering valuable theoretical and practical insights.

### Mediation role of OAT and Direct/Indirect path from HSMU to ECU

The direct association between HSMU and ECU was proved statistically significant in this study, which proved that HSMU may also contain promote-vape messages like general social media [24, 25]. Compared to previous studies [26], our contribution lies in validating the existence of this relationship among the population of current cigarette users. Furthermore, our study unveiled a significant mediating variable, OAT, through which HSMU indirectly influences ECU frequency among cigarette users.

This mediating model provides an explanatory framework for understanding the underlying mechanisms of this behavioral transition and extends to the literature in two ways. First, our results revealed that cigarette users who used health-related social media frequently were more likely to be exposed to online anti-tobacco messages. To clarify this correlation, we suggest that social media serves as a conduit for disseminating anti-tobacco messages across diverse online platforms. Cigarette users who engage with health-related social media platforms actively seek out anti-tobacco messages [23]. Online platforms use algorithms to remember user preferences [39]. They may share data with third-party developers [64], leading to the transfer of preferences from social media to other online platforms, which subsequently promotes content that is similar to that of their past searches [65]. Consequently, cigarette users are more likely to encounter anti-tobacco messages online [38].

Previous studies have shown that current cigarette users are more likely to be anti-tobacco advertisement audiences [34–36]; however, there is minimal evidence to suggest that the use of health-related media increases the chances of encountering such anti-tobacco ads online among current cigarette users. Our study proved that health-related social media are increasingly used as information sources to support individuals in behavior change attempts, including smoking cessation [66].

Second, our findings elucidated the unexpected phenomenon that current cigarette users who are more exposed to online anti-tobacco messages show a positive association with the use of e-cigarettes. This seemingly paradoxical outcome may be elucidated by considering various psychological and informational factors. Existing research, such as that conducted by Jonsdottir [67], has highlighted the unintended consequences of anti-tobacco messages on current cigarette users’ intentions to quit, such as triggering reactance among adults who smoke. Harm reduction theory also considers the difficulties of quitting risky behavior immediately and proposes using substitute behaviors to reduce harm. In the context of anti-tobacco messages, this process might manifest as an increased inclination to use other forms of tobacco products, such as e-cigarettes, as an alternative to traditional cigarettes.

Research has demonstrated that anti-tobacco messages predominantly concentrate on cigarettes, while campaigns specifically targeting the prevention of ECU are comparatively limited [40, 41]. This discrepancy in messaging emphasis may result in a lesser anti-attitude toward ECU among individuals. Additionally, the online environment is rife with diverse and sometimes conflicting information. Recent work has revealed misleading information within the online anti-tobacco content landscape, including the promotion of ECU [42]. Current cigarette users exposed to anti-tobacco messages online may encounter this misleading information, which could downplay the risks associated with ECU or present them as a viable alternative.

### Moderation role of HPE

Moreover, our research underscores the negative moderating influence of harm perception on the relationship between OAT and ECU. Individuals’ preexisting perceptions significantly influence how they respond to anti-tobacco messages, amplifying or decreasing their willingness to favor ECU. While most current research has explored the link between ECU and perceptions that ECU is safer than traditional cigarette use [44], few studies have shown that greater awareness of potential e-cigarette harm can deter ECU [68]. Few studies have focused on the context of exposure to online anti-tobacco messages or explored its potential moderating effects.

### Implication and contribution

Our findings make significant theoretical contributions to both public health and HSMU. On the one hand, this study proved the paradoxical impact of anti-tobacco messages on current cigarette users, underscoring the importance of exercising caution in the design and implementation of anti-tobacco messages and strategies to facilitate smoking cessation effectively [69–71]. On the other hand, our study further illustrates that even within HSMU, the potential for adverse health effects exists [72]. Further verification of previous studies showed that e-cigarette manufacturers frequently employ unverified health claims [73], which have the potential to mislead users [26].

The moderating role of harm perception in reducing ECU also has important implications. When confronted with anti-tobacco messages, current cigarette users with greater perceptions of harm from e-cigarettes may interpret these messages in a way that maximizes the perceived risks of ECU. This cognitive process may contribute to a decreased inclination to turn to e-cigarettes as an alternative. This alignment with Harm reduction theory suggests that accurate perceptions of harm play a crucial role in shaping individuals’ responses to ECU.

Given these findings, public health practitioners and policymakers must exercise caution in endorsing health promotion strategies to prevent unintended counterproductive outcomes. Furthermore, there is a pressing need to enhance harm education initiatives further. These programs should provide accurate and comprehensive information about the risks associated with e-cigarettes [74], particularly among current cigarette users and those considering smoking cessation.

Moreover, there is a need to ensure that harm education initiatives are accessible and engaging, reaching individuals through various channels, including social media and other digital platforms where information consumption is prevalent. By equipping individuals with a well-rounded understanding of the risks involved, harm education can empower them to make informed decisions about their tobacco and nicotine product use, ultimately contributing to improved public health outcomes.

### Limitations

This study also has limitations. First, its cross-sectional design prevents the establishment of causal relationships. Notably, the observed relationships among HSMU, OAT, HPE, and ECU are correlations. Future studies should address this limitation and explore causality using experimental methods and panel surveys. Second, the secondary data limited the scope of certain variables, such as HPE, measured with questions offering only relative harm perception. Additionally, due to the limited size of the subsample consisting of current cigarette users, we are unable to differentiate between the various exposures to cigarettes and e-cigarettes among different demographic groups. This is especially applicable to the distinction between adults and older adults (those above 64 years of age) and requires further exploration through qualitative methods such as deep interviews.

## Conclusion

This study advances our understanding of the mechanism underlying the HSMU, OAT, HPE, and ECU. The discovery of the mediating role of OAT and the moderating effect of HPE contributes to the refinement of public health communication strategies and policies. By leveraging these insights, we can foster more informed decision-making and facilitate meaningful changes in ECU behaviors among adults who smoke.

## Data Availability

The datasets generated and/or analysed during the current study are available in the open access repository, via http://hints.cancer.gov/.

## Abbreviations

ECU: E-cigarette use
HSMU: Health-related social media use
OAT: Online anti-tobacco messages exposure
HPE: Harm perception of e-cigarettes
HRT: The Harm Reduction Theory
